# The formation of the ocean’s anthropogenic carbon reservoir

**DOI:** 10.1038/srep35473

**Published:** 2016-11-03

**Authors:** Daniele Iudicone, Keith B. Rodgers, Yves Plancherel, Olivier Aumont, Takamitsu Ito, Robert M. Key, Gurvan Madec, Masao Ishii

**Affiliations:** 1 Stazione Zoologica Anton Dohrn, I-80121 Naples, Italy; 2 AOS Program, Princeton University, Princeton, NJ 08544, USA; 3 Department of Earth Sciences, University of Oxford, Oxford OX1 3AN, UK; 4 Sorbonne Universités, UPMC, Univ Paris 06-CNRS-IRD-MNHN, LOCEAN/IPSL, Paris, France; 5 School of Earth and Atmospheric Sciences, Georgia Institute of Technology, Atlanta, Georgia, USA; 6 National Oceanography Centre, Southampton SO14 3ZH, UK; 7 Oceanography and Geochemistry Department, Meteorological Research Institute, JMA, Tsukuba-city, Ibaraki 305-0052, Japan

## Abstract

The shallow overturning circulation of the oceans transports heat from the tropics to the mid-latitudes. This overturning also influences the uptake and storage of anthropogenic carbon (C_ant_). We demonstrate this by quantifying the relative importance of ocean thermodynamics, circulation and biogeochemistry in a global biochemistry and circulation model. Almost 2/3 of the C_ant_ ocean uptake enters via gas exchange in waters that are lighter than the base of the ventilated thermocline. However, almost 2/3 of the excess C_ant_ is stored below the thermocline. Our analysis shows that subtropical waters are a dominant component in the formation of subpolar waters and that these water masses essentially form a common C_ant_ reservoir. This new method developed and presented here is intrinsically Lagrangian, as it by construction only considers the velocity or transport of waters across isopycnals. More generally, our approach provides an integral framework for linking ocean thermodynamics with biogeochemistry.

High quality measurements demonstrate that the oceans have absorbed about a third of anthropogenic CO_2_ (C_ant_) emissions^1^,^2^. However our understanding of the mechanisms governing this uptake are quite elementary. The air-sea exchange of C_ant_ should be expected to play a first-order role in determining the rate of exchange of C_ant_ across the base of the ocean’s planetary boundary layer or mixed layer. This mainly occurs in the Equatorial region and mid-to-high latitudes^3^. Previous efforts such as model-data synthesis using inverse techniques^3^,^4^,^5^,^6^,^7^ have investigated meridional transport of water and C_ant_ in an Eulerian framework. This work tended to emphasize the role of C_ant_ uptake associated with the Southern Ocean divergence. Recent attention has been devoted to the relationship between anthropogenic heat and carbon uptake by the ocean^8^,^9^. None of this research to date has articulated a mechanistic framework linking ocean uptake of carbon to thermodynamic processes associated with the ocean overturning. Here we develop such a framework based on previous efforts to understand oceanic carbon uptake pathways^10^,^11^,^12^,^13^, with special focus on the shallow overturning circulation (SOC).

The SOC includes overturning of tropical waters (TW), subtropical mode waters (STMW) and subpolar mode waters (SPMW), primarily Subantarctic Mode Water (SAMW)^14^ (see Fig. 1). Observations indicate that the waters with densities characteristic of the SOC (σ < 27.0) and intermediate waters (IW; 27.0 ≤ σ < 27.5) contain as much as 63–83% of the global C_ant_ inventory, yet occupy only 27.1% of the global ocean volume (Fig. 2 and Supplementary Fig. 1, Table 1). The range depends on the method used to estimate C_ant_ concentrations at the time of WOCE^1^,^15^,^16^. To understand the higher efficiency of these water masses in retaining C_ant_ we must first understand the formation mechanism(s) of this reservoir. This exercise is particularly important considering the much shorter times scales of re-emergence into the surface layer (thereby inhibiting further C_ant_ uptake) of these water masses as compared to deeper water masses. In addition, they contribute a much lower fraction to the total C_ant_ inventory relative to what would be expected given their the large surface area of their outcrop regions (94.8%; Fig. 2, Table 1). The apparent mismatch between a large area available for air-sea exchange and a relatively small interior inventory for C_ant_ suggests that (a) air-sea C_ant_ fluxes are small in the tropics relative to high latitudes, (b) C_ant_ is exported from the lighter layers to denser water masses, or both.

We present here a novel suite of analysis tools that aim to deconvolve the roles of gas exchange, ocean interior diapycnal transports, and ocean interior diapycnal diffusive exchanges in prescribing how the ocean interior C_ant_ reservoir is formed. We identify a central role for poleward ocean transport of C_ant_, related to the poleward transport of heat associated with the SOC. This transport sustains a strong exchange of C_ant_ between STMW and SPMW, driven by near-surface diabatic processes. These diabatic processes and subsequent internal down-gradient diffusion of C_ant_ play first-order roles in controlling the routing and partitioning of C_ant_ in the ocean, and they contribute to the large subduction of C_ant_ occurring over the Southern Ocean and North Atlantic^1^.

## Basic requirements for global uptake and storage of C_ant_

Formation of the ocean’s C_ant_ reservoir is due to air-sea fluxes, ocean circulation and small-scale diffusive processes. Here we focus on those processes that maintain overturning and subduction of water masses, thereby contributing to the carbon inventory^3^. Discrete water masses, here defined as the water contained between isopycnal layers, are a macroscopic expression of oceanic adjustment to air-sea interaction in its various forms. The quasi-adiabatic behaviour of the ocean interior makes isopycnal layers the preferred horizons for studying both physics and biogeochemistry.

The complexity of the processes setting the ocean circulation can be simplified by considering the volume budget of waters in density space, i.e. the integral framework developed by Walin^17^ and extended through numerous subsequent publications^18^,^19^,^20^. In contrast to a purely kinematic account through an overturning streamfunction, this framework facilitates an evaluation of diabatic processes acting on different water masses. Access to diabatic processes allows one to link C_ant_ overturning across water masses, and finally its routing into the ocean interior to the thermodynamic processes (air –sea buoyancy fluxes, etc) that maintain the circulation. It also warrants mention that the microscale irreversible processes that diffuse C_ant_ across density interfaces are also best considered as a component of the thermodynamic controls on C_ant_. This contribution is evaluated separately.

**Figure 1:**
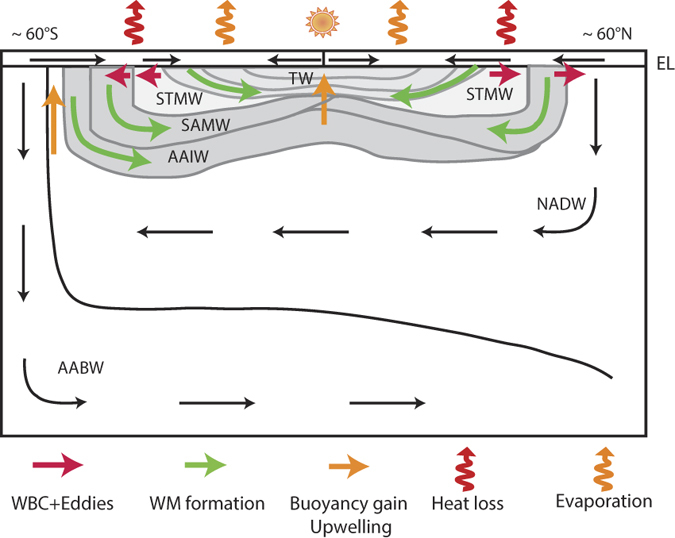
Schematic of the ocean overturning circulation (scheme adapted from ref. 35). The focus of this work is the shallow overturning circulation (SOC), spanning tropical thermocline waters (TW), subtropical mode waters (STMW), and Subantarctic Mode Water (SAMW), with the latter two water masses coupled thermodynamically. Also important in the diagram is the deep overturning, comprising North Atlantic Deep Water (NADW) and Antarctic Bottom Water (AABW). Antarctic Intermediate Water (AAIW) lies between the upper and deeper overturning structures. The Ekman layer (EL) has a significant poleward flow in the tropics which is inverted for higher latitudes. At mid-latitudes the poleward export of water is dominated by the Western Boundary Currents (WBC) and the eddy transport. In the interior, the overturning is delimited by the shallow, ventilated thermocline and the interior pycnocline, mainly set by the Southern Ocean wind-driven flow and by eddies in the Antarctic Circumpolar Current (ACC)^35^.

Our goal is thus to develop a framework that links C_ant_ uptake rates and uptake pathways to thermodynamic processes and the associated overturning circulation (Fig. 1). Given the rich complexity of the processes involved, we will proceed first using a *conceptual* model for C_ant_ inventory formation, i.e., a model that contains only those ingredients essential for representing the C_ant_ inventory formation. Our goal is to quantify the relative roles of air-sea exchange, water mass transformations, and diffusive exchanges using a streamlined conceptualization. The model is respectful of the outcrop areas and interior volumes of the observed water masses, and yet able to reproduce the observed inventory. To achieve this, we integrate elements derived from observations with information on diapycnal exchanges derived from a general circulation model.

Consider an ocean layer with volume *V*_*ρ*_ bounded by two isopycnal surfaces, *ρ* and *ρ* + Δ*ρ*, that outcrops at the surface and leaves the domain via an open boundary (Fig. 3). A net volume flux Δ*ψ* exits the domain while the diapycnal fluxes Ω_*ρ*_ and Ω_*ρ+*Δ*ρ*_ occur across the isopycnal surfaces *ρ* and *ρ*_+_Δ*ρ*, respectively. The evolution of the tracer inventory *Vc* in such a density layer, where *V* is the constant water mass volume, *c* its tracer concentration and Φ_a_ is the air-sea fluxes over the outcrop area, is given by:


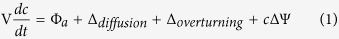


The inventory will change if the combination of the air-sea flux of the tracer, the divergence across the bounding isopycnal surfaces due to diffusive fluxes (Δ_*diffusion*_) and transport (Δ_*overturing*_), and of the exchange *c*ΔΨ with the surrounding basins is non-zero.

We now invoke Occam’s Razor in order to construct a conceptual model significantly simplified relative to those used previously to evaluate the C_ant_ distribution^21^,^22^,^23^,^24^,^25^. Consider the global ocean to consist of *n* density layers that are in steady state, internally homogenous, and exposed to an atmospheric tracer whose concentration (*c*_atm_)increases exponentially with time starting from a null value (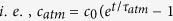), similar to C_ant_) (we do not consider here partial pressures). The simplest form of Eq. 1 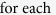
density layer *i* can be written as


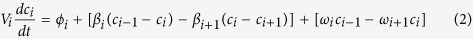


where the subscripts *i* − *1* and *i* + *1* indicate the layers immediately above and below the layer *i*, *V*_i_ is the constant volume of watermass, and *c*_*i*_ its tracer concentration and where the diabatic transport of water (i.e., the water flux across the isopycnal) is *ω*_*i*_ ≥ 0∀*i*. (See Methods for details on the assumptions). The first term on the right is the air-sea flux integrated over *A*_*i*_, the outcrop area. It is defined as *ϕ*_*i*_ = *A*_*i*_*k*(*c*_atm_ − *c*_i_), with transfer velocity *k*. Defining *H*_*i*_ as a vertical scale that can be considered equal to the annual mean thickness of the mixed layer, we get a time scale *τ*_*i*_ = *H*_*i*_/*k* (here *τ*_*i*_ and *H*_*i*_ are considered equal for all the layers). The second term (in square brackets) is the diffusive term Δ_*diffusion*_ of Eq. 1, where *β*_*i*_ is the equivalent of a volume transport across the interface between the layer *i* and the neighboring density layers *i* − *1* and *i* *+* *1*. The term in square brackets roughly mimics the convergence of a down-gradient flux of tracer *β*Δc, i.e., the diffusive transport of the tracer from tracer-rich layers to adjacent, tracer-depleted layers. The last terms represent water mass overturning, i.e., the effect of diapycnal volume transports *ω*_*i*_ and *ω*_*i+1*_ across the bounding layer surfaces. Given that the isopycnals do not necessarily coincide with the tracer isopleths it can happen that net tracer flux across a boundary is not zero even if the net water flux is. On the other hand, even if a tracer has a constant value across adjacent layers (*c*_*i−1*_ = *c*_*i*_ = *c*_*i+1*_), a water volume convergence would act to change the layer tracer inventory while the microscale turbulence that promotes the tracer diffusion would play no role. These extreme cases illustrate the profound difference between the two diabatic processes. The water volume flux is partially driven by microscale turbulence that acts to change the temperature and the salinity of the water mass, thus changing its density. Since we consider the global oceans there is no transport *c*ΔΨ outside the domain. The equations are solved numerically.

**Figure 2:**
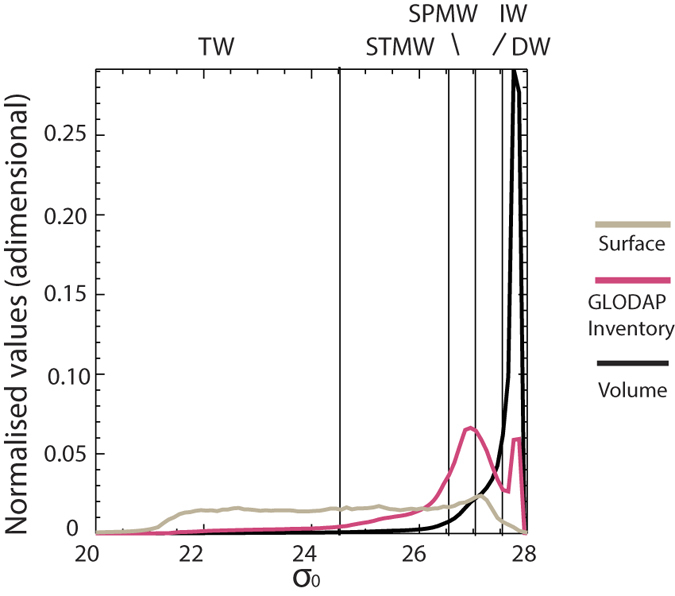
The global oceans density layering: outcrop areas, volumes and carbon inventories. Global ocean outcrop surface (grey), volume (black) in density space from monthly density fields from the World Ocean Atlas 2009^41^ and global C_ant_ inventory in density space (magenta) from GLODAP in red (ΔC*^1^). Areas under the curves have been normalised to unity. Important water mass bands are demarcated, distinguishing tropical waters (TW; 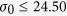), subtropical mode waters (STMW; 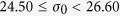), subpolar mode waters (SPMW; 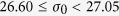), intermediate waters (IW; 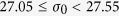), and deep waters (DW; 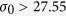).

We consider three cases of increasing complexity. In each case results are tested against the GLODAP^26^ C_ant_ inventory. In each case we simulate the ocean tracer inventory change over 200 years, with τ_atm_ = 45*yr* and *c*_0_ = 1. We choose a value of 0.1 for the density intervals and a value for *k* that gives τ = 360 *days, H* = *60* *m*. Density layer volumes and outcropping areas are taken from the observed values for the real ocean (Fig. 2, Table 1). The initial condition is a depleted ocean (i.e., c_i_ = 0 ∀*i*).

In the first case (AIRSEA) each ocean layer directly absorbs the atmospheric tracer but is isolated from the other layers (i.e., *β* = 0 and ω_*i*_ = 0 ∀*i*). This model has an inventory (Fig. 4a) that resides mostly in subpolar and intermediate waters, thus capturing one of the main observed features. Furthermore, the highest air-sea fluxes are into intermediate water masses (Fig. 4d).

Insights can be gained through closer inspection of the details of the solution. This case admits an analytical solution (see Methods) that can be approximated, for *e*^t/τatm^ ≫ 1, as *c*_*i*_ = *γ*_*i*_*e*^t/τatm^, where 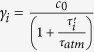, with 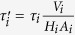 (i.e., the relaxation time is modulated by the ratio between the total volume and *H*_*i*_*A*_*i*_ the volume of the boundary layer of the water mass). In the case of the rather shallow subtropical and tropical waters 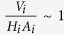 and thus, given that 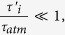 *γ*_*i*_ ~ *c*_0_. In this case the inventory closely follows the atmosphere. For deep waters, since 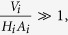 the opposite occurs, i.e., *γ*_*i*_ ~ 0 and consequently *c*_*i*_ ~ 0.

The air-sea fluxes, 
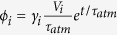
, and the inventory, *V*_*i*_*c*_*i*_ = *V*_*i*_*γ*_*i*_e^t/τatm^, are both proportional to *V*_*i*_*γ*_*i*_. Since the volume *V*_*i*_ increases with density while *γ*_*i*_ decreases with increasing 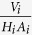 (i.e., with increasing density) this product has a peak for intermediate waters. The result is a match between the analytical solution for a motionless ocean and GLODAP that is very good up to σ_0_ = 27.2, i.e., to 75% of the observed inventory (Fig. 4d). This peaked distribution is independent of time (its structure, *V*_*i*_*γ*_*i*_, remains constant) while the inventory increases exponentially. In other words, the importance of SPMW and IW relative to global budgets does not depend upon time, thus fixing the internal turnover to their renewal time scales. The present proportion among water masses is thus not a transient despite the strong transient nature of the forcing. Finally, we note that the exponential nature of the solution explains why we found the GCM inventory and its rate of change to be proportional, as presented in the following.

Nevertheless, in the AIRSEA case, deep layers are largely depleted of tracer. By construction, the cumulative (in time) air-sea flux distribution (Fig. 4d) is the same as the inventory when both are viewed cumulatively with respect to density. By adding diffusion (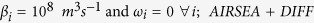) the denser water tracer content (σ > 27.4) has a better match with GLODAP (Fig. 4b), due to the internal transfer of tracer from intermediate waters to the deep layers. Nevertheless, the inventory is largely made of intermediate waters. Further, a difference between cumulative air-sea fluxes and the inventory (Fig. 4e) occurs only for the densest waters (a consequence of the internal transfer described above). Altering the diffusive constant (*β*_*i*_) does not significantly alter the outcome, as in contrast to the overturning diffusion acts only between layers with different tracer concentrations. As light waters (i.e., in the tropical and subtropical regions) are nearly saturated and since we ignored the effect of temperature on saturation, concentrations are homogenous for these waters and a gradient occurs only between intermediate and deep waters.

The third case 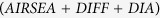 incorporates the effect of the diapycnal transport overturning. This case requires evaluation of cross-isopycnal transport by oceanic circulation, here derived from a GCM simulation (see next section). To evaluate the impact of diapycnal transport on inventories and air-sea fluxes, we consider a density layer that has no exchange with the lighter layers yet loses tracer via diapycnal transport toward denser layers (with zero diffusion), i.e., where β_i_ = β_i+1_ = 0 and ω_i_ = 0 and ω_i+1_ = const, the latter thus making the divergence of the diapycnal transport (ω_*i*_ − ω_*i*+1_).

In this simple case the tracer increase for each water mass is still exponential, but has a different shape factor:


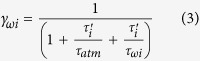


where τ_ωi_ = V_i_ ω_i+1_, is the water mass renewal or turnover time due to the divergence of the diapycnal transport. Evidently, if *τ*_ω*i*_ ≤ τ_atm_, i.e., if the water mass turnover by the overturning is faster or comparable with the atmospheric exponential increase, the inventory formation will be impacted by the former process.

Importantly, in this case


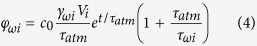


Therefore, as a consequence of the term on the right in parenthesis, the relative importance of the flux of a given water mass might be significantly increased in the presence of a fast overturning rate. This result is not surprising. It also anticipates a possible mismatch between the air-sea fluxes and the inventory in correspondence of the water masses having high turnover rates and being impacted by a significant overturning into denser waters, as is the case for the SOC.

It is easy to demonstrate that for ω_i_ > 0, φ_ωi_ > φ_i_. That is, in the presence of export of water towards denser water classes, the air-sea flux adjusts, causing increased oceanic uptake. Similar arguments can be used to show that the flux will decrease correspondingly in presence of a net overturning convergence.

## Global diapycnal C_ant_, volume fluxes and formation of the C_ant_ inventory

In order to characterize the role of the oceanic overturning, we first introduce a more comprehensive, exact formulation of the carbon inventory time evolution per water mass and evaluate the magnitude of the different terms in this formulation using a General Circulation Model (GCM). We then investigate the limits of validity for the conceptual model described in the previous section using a GCM-based evaluation of the carbon transport due to overturning and gauge the specific role of SOC in determining the observed C_ant_ inventory.

**Figure 3:**
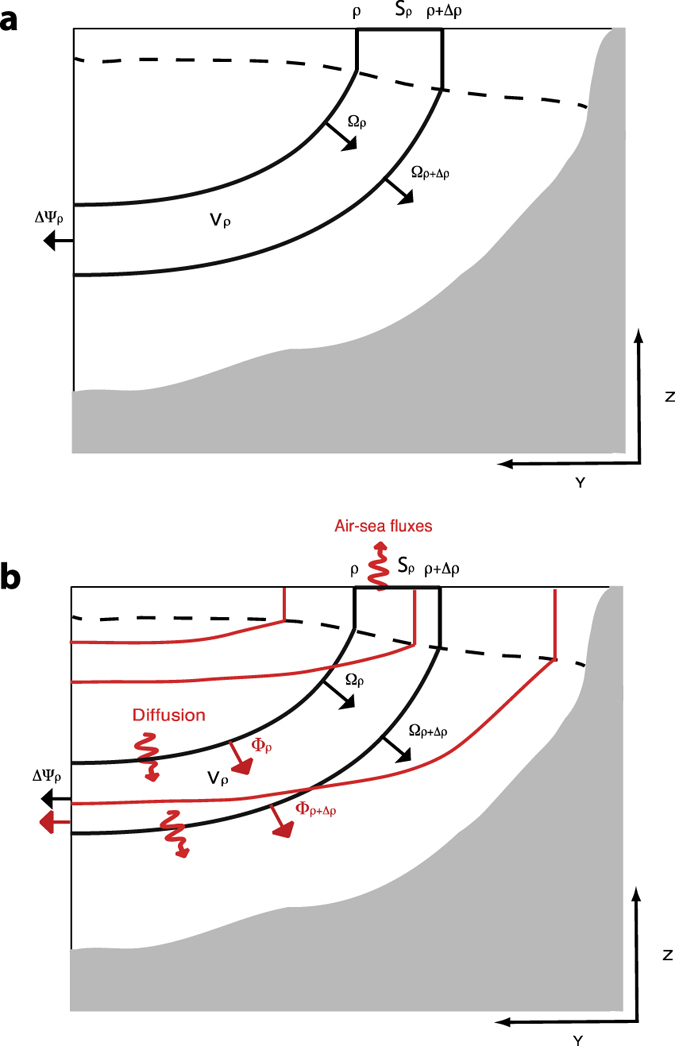
Schematics of the water masses formation in an idealized oceanic basin. Here namely a subdomain of the global ocean with an open boundary. (**a**) Volume budget. Following standard definitions we denote as *V*_*ρ*_ the volume sandwiched between the isoneutral surfaces *ρ* and *ρ* + Δ*ρ*, the ocean lateral boundary and the ocean surface. 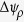 is the net volume flux of fluid exiting the domain *Vρ* across the open boundary; Ω_*ρ*_ and 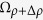 are the total volume fluxes across the isoneutral surfaces *ρ* and 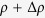, respectively (i.e. the diapycnal volume flux, defined as positive when toward denser waters (or overturning; units are Sverdrup (Sv) where 1 Sv = 10^6^ m^3^/s)). (**b**) Tracer budget for *V*_*ρ*_. Red lines indicate the tracer isosurfaces (i.e., surfaces on which the tracer is constant), which are not necessarily aligned with the isopyncals. The rate of change of the tracer inventory C in *V*_*ρ*_ is given by the sum of the diapycnal transports 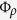 and 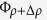 across the isoneutral surfaces ***ρ*** and ***ρ*** + Δ***ρ***, respectively, the air-sea fluxes across the layer outcrop surface 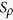, the diffusive transports across ***ρ*** and ***ρ*** + Δ***ρ*** and the tracer transport by the flow 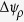 across the lateral boundary (lateral red arrow). The sign convention is such that all the fluxes are positive if toward denser layers. The dashed line indicates the base of the mixed layer. The fluxes are computed over the entire isopycnal. In the main text the diapycnal fluxes of volume and tracer are discussed separately for the mixed layer (MWML) and for the interior.

Consider again the isopycnal layer (Fig. 3) with the same diapycnal fluxes Ω*_ρ_* and Ω_*ρ*+Δ*ρ*_. (Note that it is also defined as G^18^ or A^19^). The net water mass *formation* rate is defined as the rate of accumulation *M*Δ*ρ* of the water with density 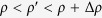 where *M* is the water mass formation per unit of density. It is calculated as the time variability of the volume (inflation/deflation) plus the flux entering or exiting the domain Δ*ψ*:


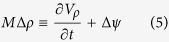


Conservation of volume requires that *M*Δ*ρ* balance the convergence of the diapycnal fluxes (Fig. 3a), i.e.,


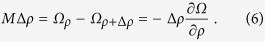


The net diapycnal *volume* transport (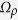; the physical overturning or water mass *transformation*^17^; here in the formulation of ref. 20) is given by the sum of the processes acting on density (the diabatic processes related to the ocean thermodynamics) (see Supplementary Material for details on the formulation). The first is the divergence of mixing processes (*d*_*p*_). This includes microscale diapycnal mixing, - lateral mixing in the mixed layer where isopycnal are nearly vertical. Mixing along the isopycnal, due to mesoscale and submesoscale eddies and filaments in the interior, diabatically impacts the water mass by causing a net transfer of water due to non-linearity of the equation of state (cabbelling, and, when using neutral density, thermobaricity). The second contribution comes from the boundary buoyancy forcing (*f*_*p*_), i.e., the various surface heat fluxes and the geothermal heating acting on temperature and the freshwater flux due to evaporation, precipitation, ice production and melting and river runoff.

In practice the divergence of mixing processes (*d*_*ρ*_) and buoyancy forcings (*f*_*ρ*_) are identified by diagnosing and combining the equivalent terms for temperature and salinity tendencies. In fact it can be proven^17^,^20^ (see Supplementary Note) that:


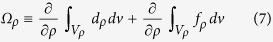


where the integrals are evaluated over the volume sandwiched by the isopycnal layer *ρ*, the ocean bottom and the basin open boundary. Positive *transformation* values indicate transports toward denser waters^17^,^18^,^19^,^20^,^21^. Here we use potential density (σ_0_) to define the reference frame.

We next extend this density-based volume budget framework by incorporating oceanic tracers. Consider the dynamical budget of a passive tracer within *V*_*ρ*_ (bounded by two isopycnal surfaces, *ρ* and +Δ*ρ*) as presented in Eq. 1 but for the full time-varying general case (i.e., a time varying water mass volume; see Supplementary material for the case of active tracers). For a passive tracer *c* (the same as above), the equation for the rate of inventory change, *C*, can be written (see Fig. 3b and Supplementary Note^21^):


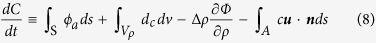


As in Eq. 1 here the first term on the rhs is contribution of the air-sea exchange (the integral over the outcrop of the air-sea fluxes per unit area *ϕ*_*a*_) and the second term is Δ_*diffusion*_, the net diffusive flux across the boundaries of 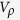 (equivalent to the integral over the volume of the convergence (*d*_*c*_) of the tracer diffusion). The third term is again Δ_*overturning*_, the convergence of the tracer flux (Φ_*ρ*_) due to the diapycnal volume transport the overturning across the bounding isopycnal surface (i.e., by the diapycnal volume transport as defined above). The last term is the tracer transport at the basin boundary, where **u** is the velocity at the boundary, c is the tracer concentration, and **n** the unit vector perpendicular to the boundary.

For a given *ρ*, the diabatic tracer transport across the isopycnal (Φ_*ρ*_) is evaluated^21^ by quantifying the thermodynamic drivers of the overturning (the same as in Eq. 7). Specifically, the diapycnal tracer transport, simplified as the product between the diapycnal transport *ω* and *c* in Eq. (2), is due to the combined variation of *c* and the constituents of the physical diapycnal transport in Eq. (7) along the isopycnals:


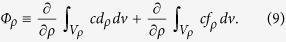


where the integrals are again evaluated over the isopycnal layer volume. In other words, the tracer transport is expressed as the sum of *all* the physical processes sustaining the overturning, from solar radiation and evaporation to mixing due to wind or to cabbeling. Eq. (9), and consequently Eq. (8), include a large number of terms derived by using salinity and temperature tendencies to build Eqs 3 and 5. Since the equation is linear, each term can be evaluated separately. As *C*_*ant*_ is a passive tracer, these equations and concepts can be used to quantify specific processes that influence formation of the *C*_*ant*_ inventory. The same argument holds for any similar biogeochemical tracer. As reported in the Supplementary text, this equation has the same properties as the approach developed by Walin for the volume flux computation. It is intrinsically Lagrangian since it naturally follows isopycnals, i.e., it considers only the velocity of the water relative to the isopycnal). It considers all possible cases, including the *C*_*ant*_ exchange across isopycnals that occurs when the sun warms the stratified region below the mixed layer *even* in absence of any water movement. In this latter case the radiative warming is de facto moving down the isopycnals creating a net diapycnal flux.

By introducing the tracer diapycnal flux due to buoyancy-altering processes Φ_*ρ*_ (Eq. 9), we introduce the concept of tracer transformation associated with water mass transformation. In addition, using Eq. 2, the tracer diapycnal transport associated to the transformation becomes an explicit term in the dynamical balance for the inventory. Note that near-surface buoyancy gradients do not strictly co-vary with *C*_*ant*_ gradients, therefore the *transformation* of volume and the associated *C*_*ant*_ transport may differ in magnitude and by region.

Water mass formation is generally associated with transformations that occur in the mixed layer^19^. There, water mass properties are set by air-sea interactions and the largest diapycnal fluxes occur. Here we use a generalization of the concept of water mass formation since here we will evaluate, independently, the transformations occurring in the mixed layer and in the interior. Specifically, to differentiate subsurface from surface processes, we evaluated the change in diapycnal volume and C_ant_ fluxes that only occur in the upper maximum winter mixed layer (MWML). Importantly, in this case the net water mass *formation* coincides with net *subduction*^27^,^28^,^29^. This is why the MWML is a common reference horizon in physical and biogeochemical studies (e.g., in the concept of preformed nutrients and carbon^30^).

### Diagnosing connections between the C_ant_ inventory, air-sea fluxes and diapycnal volume fluxes

The physical model used here is the global ORCA2-LIM^31^ ice-ocean model with nominal two-degree global resolution and isoneutral mixing (see Methods). The model was spun up for 1500 years with repeating seasonal forcing fields^20^,^21^,^29^. Bi-weekly means of the pertinent physical state variables were saved over the final year for analysis and to drive the biogeochemistry model. A twenty-four compartment biogeochemistry model, PISCES (Pelagic Interaction Scheme for Carbon and Ecosystem Studies)^32^, was spun up offline using the physical state over 3000 years with pre-anthropogenic boundary conditions. In 1860 the run was split into a control with the same pre-industrial atmospheric concentrations and another with the observed transient atmospheric CO_2_. Following standard nomenclature^6^, we refer to the first as “natural” carbon, the second as “contemporary” carbon, and the difference as “anthropogenic carbon”.

By 1995, nearly 80% of the modelled C_ant_ had accumulated at densities shallower than σ_0_ < 27.55, encompassing the SOC and IW (Table 2, Figs 1 and 2). Up to 34% of the total C_ant_ inventory was found in tropical and subtropical waters (σ_0_ < 26.6). The model distribution of C_ant_ is in good agreement with observational estimates (Tables 1 and 2; Fig. S2) having most accumulation in mode and intermediate waters but these waters are too cool in the model (a shift to denser values is observed) and the deep inventory is generally underestimated. 96% of the total air-sea flux occurs over SOC + IW outcrops and 57% (1.17 PgC/yr, Table 2, red line in Fig. 5) occurs over tropical and subtropical water outcrops. For these shallow water masses the relative importance of air-sea fluxes is greater than the relative fraction of the yearly C_ant_ accumulation rate (Table 2, blue line in Fig. 5; 96% vs 80%, or 57% vs 34%). This mismatch between C_ant_ uptake via gas exchange and interior accumulation implies a net transfer of C_ant_ to higher densities subsequent to initial uptake.

The underlying mechanisms for this transfer are explicitly quantified here using the thermodynamic water mass transformation framework introduced above. Evaluating Eq. 8 for each water mass gives the dynamics of the formation of the corresponding C_ant_ inventory (Table 2). To provide an overview of the role of the different processes we discuss first the cumulative quantities for the different terms. For a given density *ρ*, evaluating Eq. 8 from 0 to *ρ* we have the rate of change of the inventory for the layers 0 < *ρ*′ < *ρ* given as a function of the air-sea interaction, diapycnal diffusion and transport of C_ant_ across *ρ* and transport at the basin boundary (here zero since we integrate globally). As shown in Fig. 5, for a given density *ρ* a large fraction of modelled rate of change of the C_ant_ inventory (left term in Eq. 8; blue line) can be explained by subtracting the diapycnal carbon fluxes **Φ** (orange line) _**ρ**_ from the air-sea fluxes of C_ant_ (first term on right in Eq. 8; red line) using Eq. 8. Note that the diapycnal volume transport generally promotes a transfer of C_ant_ from light to dense waters. The convergence of the diapycnal C_ant_ fluxes in a layer bounded by two isopycnal surfaces (the *formation*) gives an estimate of the net contribution of the physical overturning to the layer inventory of C_ant_. Quantitatively, the diapycnal fluxes inject 0.12 PgC/yr into STMW, 0.12 PgC/yr into SPMW and 0.19 PgC/yr into deeper waters (Table 2c).

We show here that the mechanisms that promote ocean ventilation are: a) subsurface transport toward extra-tropical latitudes and then subduction (i.e., the net diapycnal convergence of C_ant_ in the MWML); b) the diffusive C_ant_ transport resulting from the diapycnal (mostly vertical) C_ant_ gradients below the MWML. The latter process is the residual between the blue and the orange lines in Fig. 5 (difference of air-sea fluxes and diapycnal formation fluxes Equation 8; Table 2d,e). Diffusion exports C_ant_ away from light, shallow waters into the intermediate and deep waters (Table 2e). The diffusive flux divergence peaks at 0.18 PgC/yr in STMW (Table 2e) and exceeds diapycnal formation fluxes for that layer (0.12 PgC/yr).

Contrasting formation fluxes for the entire ocean and the MWML (Table 2c) emphasises the importance of surface and interior water mass transformations (**Φ**_**ρ**_) for C_ant_ storage. In fact MWML fluxes are greater than fluxes into the SPMW density classes (by 0.11 PgC/yr; Table 2c). The diapycnal transport of C_ant_ out of TW is also more important than the total fluxes in that density class; −0.47 PgC/yr, or about 26% of the global air-sea flux, vs. −0.36 PgC/yr (Table 2c). Negative values indicate removal of C_ant_ through water mass *erosion* and a transfer of C_ant_-laden waters into waters of other density classes. Once air-sea fluxes and diapycnal fluxes due to water mass transformations in the MWML account for most of the inventory change for tropical and subtropical water masses (Fig. 2b). In other words, the surface diapycnal fluxes of the SOC and the air-sea fluxes sustain a C_ant_ distribution that is close to the actual inventory change. In the interior, overturning moves C_ant_ into lighter waters while diffusion does the opposite. The two mechanisms nearly cancel for layers that are approximately C_ant_-saturated and thus in near steady-state.

By construction with our cyclo-stationary physical state for the ocean, the water mass volumes do not change, and thus there is no net water mass volume inflation (i.e., net convergence of diapycnal transports). Therefore the interior processes that sustain the overturning of the water masses compensate the diapycnal transport occurring in the MWML (Fig. 1). In the latter layer surface buoyancy fluxes tend to densify the water masses. In fact in the interior the buoyancy diffusion and the penetration of solar radiation below the MWML^20^ *generally* move waters from dense layers to lighter ones (driving an *upwelling*). This diapycnal volume transport (or upwelling) in the interior would have no effect upon C_ant_ if the interior layers were depleted in C_ant_. The presence of C_ant_ in the interior waters is such that they have an effect (by re-entraining C_ant_ into the mixed layer), but their contribution is largely overwhelmed by the diapycnal divergence in the MWML. Finally, the overturning of C_ant_ associated with DW formation is found to be significant (0.19 PgC/yr; mostly NADW formation, see below), and leads to a progressive densification and enrichment of C_ant_ in DW source waters while moving from the subtropics to the high latitude formation sites.

In summary, the MWML component of the overturning dominates the diapycnal fluxes **Φ**_**ρ**_ of C_ant_ and consequently shapes the interior C_ant_ distribution. A closer look shows that in the MWML, transformation-related diapycnal transport of C_ant_ is associated with the upper poleward branch of the SOC in which light waters are converted into mode and intermediate waters (Fig. 6). The MWML volume transformation reaches a maximum across σ_0_ = 26.4 (Fig. 6a). This peak densification transfers 0.4 PgC/yr (Fig. 6b) of C_ant_ into denser water masses. This rate is about 20% of the global C_ant_ uptake for the model (2.07 PgC/yr in 1995, Table 2a). The peak C_ant_ transformation is not co-located with the peak volume transformation (Fig. 6a), but rather occurs at much lighter density due to the higher C_ant_ content of those waters.

The diapycnal C_ant_ transport towards denser waters within the MWML is quite symmetric for both hemispheres in waters with σ_0_ < 26.6 and poleward of 30° (Fig. 6b). Subtropical sources inject C_ant_ into denser classes at a maximum rate of nearly 0.3 PgC/yr. At higher densities in the Northern Hemisphere, a peak for NADW formation (σ_0_ ~ 27.7; 0.2 PgC/yr) is evident (Fig. 6a,b). This process is fed by overturning more than local air-sea C_ant_ fluxes (Fig. 6b). Neither volume nor C_ant_ fluxes scale as well in the Southern Ocean. Bottom water formation in the Antarctic is not well resolved in potential density space^20^, nor does it have a large impact on the model C_ant_ inventory (Fig. 6b). Upwelling and transformation of CDW into SAMW in the Southern Ocean is clearly visible as negative values in Fig. 6a (~10–15 Sv), but these are not associated with a significant C_ant_ transport (magenta arrows, Fig. 6a,b). The upwelling carbon-depleted CDW increases the air-sea fluxes by increasing the difference in pCO_2_ between the atmosphere and the ocean. Importantly the supply of C_ant_ to SAMW by the water mass overturning is dominated by the contribution from the subtropics (i.e., by the SOC). In the tropics the diapycnal C_ant_ transport is also very different from the diapycnal volume transport (σ_0_ < 24.5, green arrow, Fig. 6b) due to lower C_ant_ content of newly obducted waters.

Overall, the upper branch of the global overturning (that occurring mostly in the MWML) is thus exporting C_ant_ to mid and high latitudes. It is important to reconcile this result with the current estimates of the meridional transport of C_ant_. The present consensus is that globally C_ant_ converges toward the Equatorward (except for the North Atlantic^2^,^16^). This contradiction is only apparent. By invoking a water mass framework, we are here evaluating the C_ant_ that is redistributed by the overturning and not on the vertically integrated (meridional) transport. As illustrated with the conceptual model, a large component of C_ant_ stays in the isopycnal layers in which it entered via air-sea fluxes. In other words the meridional transport is being mostly sustained by flow *along* isopycnals (i.e., the adiabatic component of the flow). Given that the air-sea fluxes of C_ant_ are maximal for subpolar mode and intermediates waters, and as these water masses in the interior exhibit a net flow from the high and mid-latitudes towards the tropics, they provide most of the meridional transport. Importantly, in the North Atlantic the poleward transport of C_ant_ in the upper layer by the surface circulation associated with the NADW formation is such that it dominates the meridional transport locally. The role of the overturning considered here is consistent with the view presented in ref. 33, although their kinematic approach did not allow for an explicit connection to ocean thermodynamics.

An important benefit of a thermodynamic-based water mass framework to C_ant_ (Eq. 9) is that it allows for identification and quantification of all mechanisms that control the transfer of C_ant_ into the ocean interior. The air-sea exchange of buoyancy sustains a net flux towards higher densities for waters denser than σ_0_ = 24.0 (peak of 0.5 PgC/yr at σ_0_ = 25.0 in Fig. 7a). Similarly, atmospheric buoyancy fluxes transfer C_ant_ (0.6 P gC/yr) toward waters lighter than σ_0_ = 22.0. The densification of tropical waters is evenly distributed between the two hemispheres (Fig. 7a, σ_0_ = 24.5). The densification of light tropical waters during their poleward journey is largely sustained by net evaporation (peak at σ_0_ = 23.5 in Fig. 7b). Cooling dominates transformation at higher densities (Fig. 7b), but the both processes are needed to sustain the C_ant_ export to STMW and SAMW. The transfer towards light (tropical) waters is due to the combined effects of penetrative solar radiation (up to 0.8 PgC/yr at σ_0_ = 23.0) and freshwater input, centered at σ_0_ = 21.5 (effectively the Warm Pool). The net transfer of C_ant_ from the subtropics to the Southern Ocean by buoyancy forcing is balanced by the local net gain of freshwater at the surface (negative peak at σ_0_ = 27.3, Fig. 7b). In the Northern Hemisphere, high C_ant_ fluxes into NADW are associated with cooling of subtropical waters that have accumulated anthropogenic C_ant_ while moving poleward. Categorizing evaporation and cooling as the main drivers of poleward transport of C_ant_ holds even when considering mixing, which also influences heat and freshwater budgets (Fig. 7c). However, the surface heat and freshwater fluxes that induce a transport towards the lighter waters across σ_0_ = 22.0 (Fig. 7c) are overcompensated by mixing (Fig. 7c). This is more clearly visible in Fig. 7d. The large surface input of heat and freshwater that promotes the formation of light waters is more than compensated by the (mostly vertical) mixing in the tropical and subtropical part of the SOC. In the SOC, mixing is the thermodynamic counterpart of the Equatorial upwelling and surface divergence. Mixing is, however, subordinate to the release of heat to the atmosphere in further densification of water parcels along the poleward journey at the surface. This holds at least up to the higher water densities, σ_0_ = 26.9–27.3, where mixing processes again contribute significantly.

## A conceptual model of the global uptake and storage

Finally we return to the conceptual model to see if the addition of diapycnal C_ant_ transport from subtropical to intermediate and dense waters derived from the GCM significantly improves the solution there. If that were to be the case, then we would have identified the main drivers in forming the reservoir. In other words, the conceptual model’s elements (water mass aspect ratios and the presence of a shallow overturning) are sufficient to capture the important dynamics.

We assume here that only the shallow component of the overturning is relevant. Specifically, we use the values for *ω* given from the ocean circulation model (Fig. 6a; Methods) for the export of water from the tropics toward the subtropical and subpolar regions.

**Figure 4:**
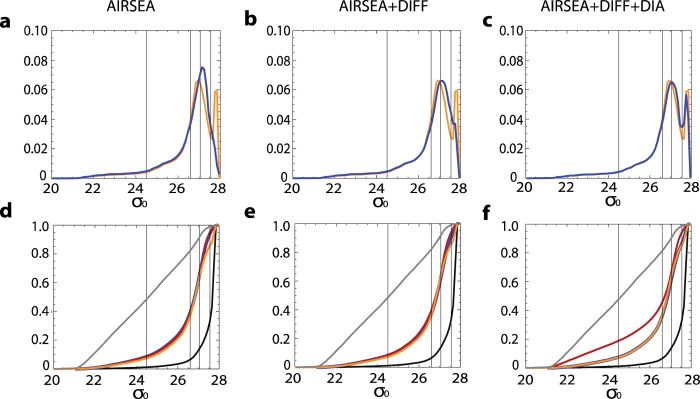
Results from the conceptual model. Here the inventory obtained with conceptual model are compared with GLODAP. First we consider the AIRSEA case (**a**) with the GLODAP inventory in orange and in blue the inventory predicted by the numerically integrated conceptual model (Eq. 2). Both inventories integrals were normalised to 1 to ease comparison. The analytical solution presented in the text has not been superimposed since it coincides with the numerical one. (**b**) Same as (**a**) but for AIRSEA + DIFF case. (**c**) Same as (**a**) but for AIRSEA + DIFF + DIA case. The cumulated terms of the Eq. 2 are also presented, with each cumulated variable being normalised to unity. The case AIRSEA is shown in (**d**) with in blue the rate of change of the inventory (blue) and in red the carbon air-sea fluxes. For each density value these are thus the estimates of the corresponding rates of change occurring for the layers having lower or equal densities. The cumulated GLODAP inventory (orange), WOA outcrop areas (grey), WOA layer volumes (black) are also shown. The color scheme is the same in Fig. 5, where the equivalent GCM estimates are presented. (**e**) the same as (**d**) but for the AIRSEA+DIFF case. (**f**) the same as (**d**) but for the AIRSEA + DIFF + DIA case.

**Figure 5:**
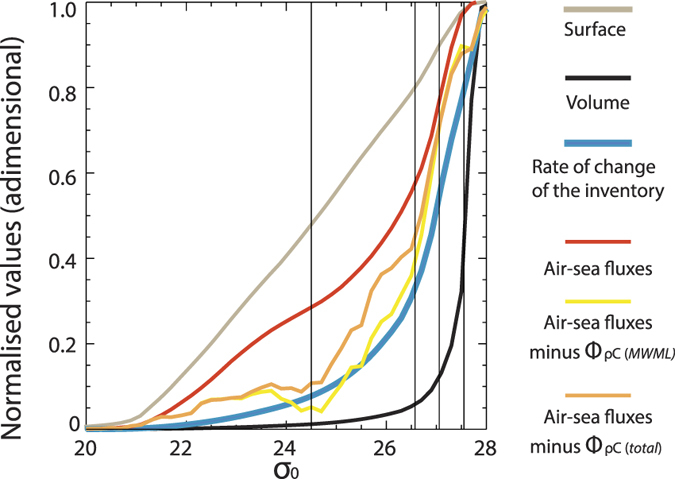
Global carbon budgets. Model global estimates of the terms in Eq. 8. Integrals in Eq. 8 are performed cumulating from 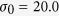. Grey: Integrated outcrop area (i.e., for each density value 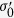 the area where 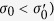; Red: Integrated uptake of anthropogenic carbon by gas exchange during 1995; Blue: Integrated rate of interior accumulation during 1995 (Note: in fact, it is very similar to the integrated global model inventory of C_ant_); Black: Integrated volume of the ocean density layers. The difference between the red and the light-blue curve illustrates the importance of processes other than the air-sea fluxes setting the inventory. Additionally, the yellow curve represents the air-sea fluxes minus the diapycnal transports of C_ant_ in the MWML (Fig. 6b; Eq. 8) while the orange curve represents the air-sea fluxes minus the total diapycnal transports (first and last terms in Eq. 8). The difference between the latter and the inventory is due to diffusive C_ant_ fluxes.

**Figure 6:**
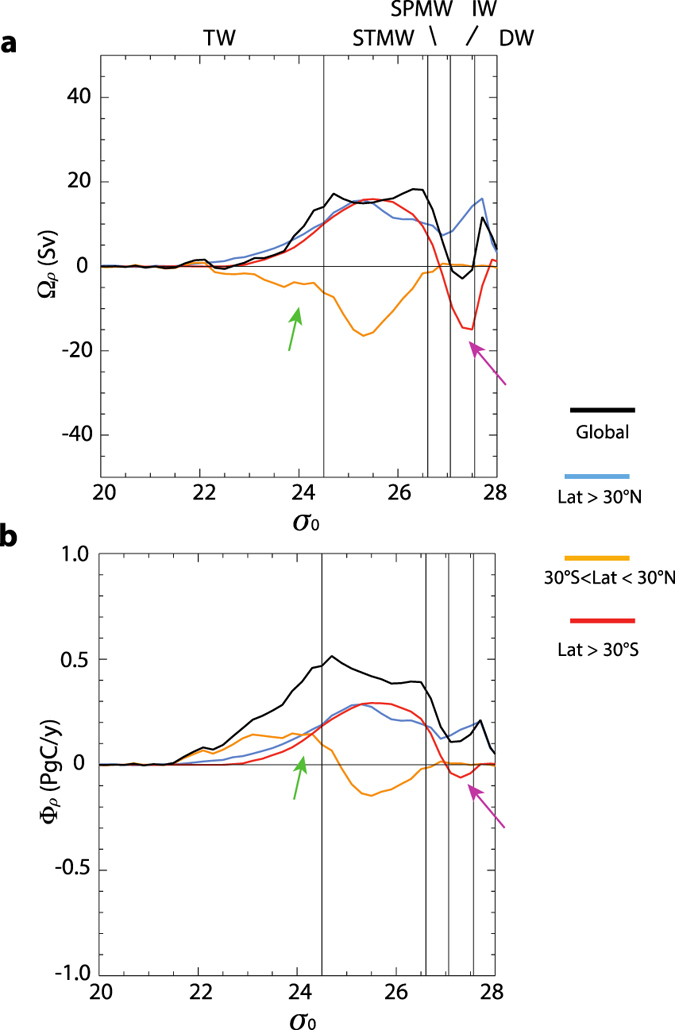
Net MWML diapycnal transports. (**a**) Volume (Ωρ) and (b) C_ant_ (Φ_*ρ*_) *transformation* fluxes in the MWML due to all processes over the density range 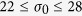. In (**a–b**), oblique magenta arrows underline the large difference between volume and C_ant_ transports associated with the upwelling of CDW. The green arrow points to the density range showing the greatest difference between heat and C_ant_ transformation fluxes, the latter having a much smaller (but not negligible) re-emergence at the Equator. Line colors show global (black) and latitude-separated results: North of 30°N (blue), between 30°N and 30°S (orange), south of 30°S (red). In all panels, positive (negative) *transformation* values represent diapycnal transports towards denser (lighter) densities. The slope of the lines represents the *formation*: convergence (negative slope) and divergence (positive slope). Values for σ_0_ < 26.4 were smoothed with a 3-points running mean filter.

**Figure 7:**
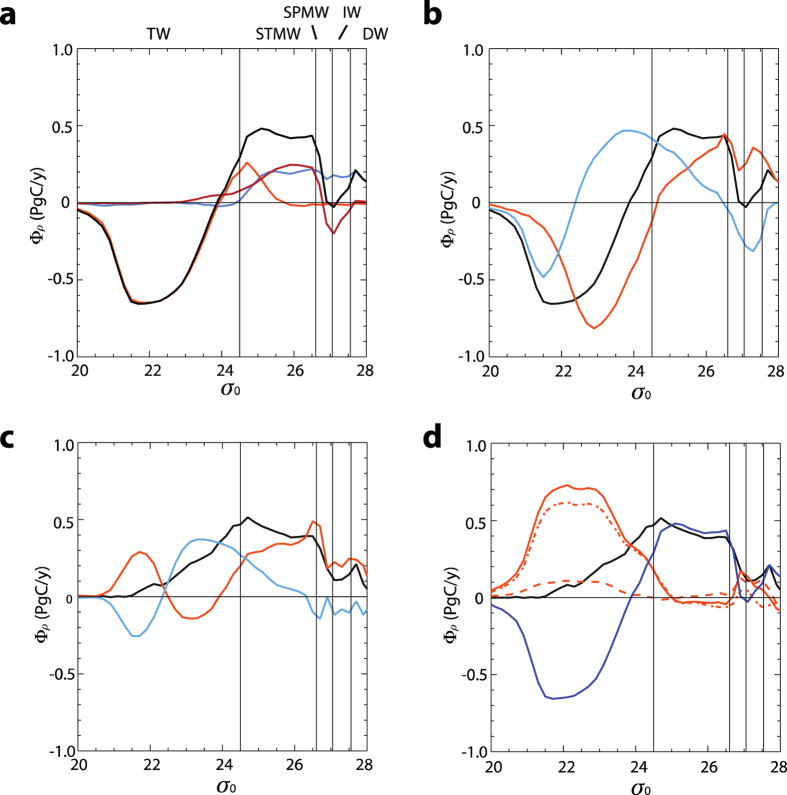
Details of the processes sustaining the carbon overturning. (**a**) Carbon transformation fluxes (Φ_*ρ*_) in the MWML due to surface buoyancy fluxes only. Line colors show global (black) and latitude-separated results: North of 30°N (blue), between 30°N and 30°S (orange), south of 30°S (red). (**b**) Same as (**a**) (black), showing also the decomposition into the global freshwater (blue) and heat (red) components. (**e**) Same as (6b) (black), showing also the decomposition into the global freshwater (blue) and heat (red) components. (**d**) Same as (**c**) (black), showing the decomposition into surface buoyancy fluxes (blue same as (**a**)) and buoyancy mixing fluxes (solid red line). The dashed red line represents the effect of the lateral mixing processes while the dot-dashed red line indicates the effect of vertical mixing processes. In all panels, positive (negative) transformation values represent diapycnal transports towards denser (lighter) densities. The slope of the lines represents the formation: convergence (negative slope) and divergence (positive slope). Values for σ_0_ ≤ 26.4 were smoothed with a 3-points running mean filter.

The addition of overturning makes a dramatic difference. First, the bimodal distribution of the GLODAP C_ant_ inventory is now well reproduced (Fig. 4c; Methods), with a separated peak for the deep reservoir. This is formed by densification of C_ant_-rich water rather than by local air-sea fluxes and it reproduces the importance of NADW formation to the total C_ant_ inventory. In addition, the peak for the lighter water masses is now more correctly centered between SPMW and IW rather than on IW. There is only now a strong separation between the cumulative (in time) air-sea fluxes and inventories, when both are viewed as cumulative quantities with respect to density (Fig. 4f), as the air-sea flux component has now shifted towards lower densities. Stated differently, it is only now with the explicit inclusion of the overturning component that the conceptual model is able to match the results obtained with the ocean circulation model (compare Figs 4f and 5).

The conceptual model here has considerable skill in reproducing the C_ant_ inventory and its relation to the air-sea fluxes. It provides mechanistic insight in three ways. First, it illustrates how the aspect ratio of the ocean layers, that is set by the oceanic response to wind and buoyancy fluxes^24^, is the dominant factor, with IW having the optimal geometry. Second, it illustrates the complementary roles played by tracer diffusion across isopycnal surfaces and the overturning circulation. In fact, while diffusion is important, it is the overturning that guarantees a flushing out of the tropical and subtropical regions in favour of mode and IWs, thereby sustaining enhanced uptake via gas exchange at low latitudes. Third, only the dense overturning component (σ_θ_ > 27.5) can account for the deep C_ant_ reservoir since these waters are weakly influenced by air-sea exchange. This transfer of C_ant_ into the densest waters creates an inventory gap between mode/intermediate waters and deep waters.

To summarize, the conceptual model underscores the central role of the shallow overturning circulation (SOC) in determining the interior inventory of C_ant_. The predicted inventory is sensitive to details of the imposed overturning (see also ref. 24). This exercise illustrates the importance of understanding the mechanisms responsible for the overturning circulation and the sensitivity of that circulation to various perturbations, including the response to climate change.

## Discussion

The framework presented here provides a means to reconcile the disparate density signatures of C_ant_ uptake by gas exchange (considered cumulatively in time) and C_ant_ accumulation in the ocean interior. As such, it is our hope that the methods can be applied more generally to the synthesis of surface carbon products (SOCAT) and ocean interior products (for instance, data comprised by GO-SHIP, or GLODAPv2 34). In fact the method is quite general, and can be applied to interpret any biogeochemical tracers that are impacted by the overturning circulation of the ocean.

We set out to test the hypothesis that ocean overturning and diffusion strongly modulate the pathways by which C_ant_ enters the ocean interior, and we developed a schematic view whereby C_ant_ “rides” or participates in the overturning structures associated with the poleward transport of heat by the ocean^35^. To test this idea, we applied a thermodynamic water mass framework^17^ to the case of C_ant_. Previous studies have invoked overturning and water mass concepts to interpret C_ant_ uptake^1^,^3^,^13^,^33^ but these have not explicitly considered the ensemble of physical processes involved in regulating the uptake of C_ant_ by the ocean. Here, the joint use of the simplified conceptual model and the full three-dimensional OGCM provided a means to deconvolve the mechanistic controls on C_ant_ uptake pathways into the ocean.

The ocean C_ant_ inventory is strongly influenced by water mass transformations and diffusion, thereby reflecting the large-scale overturning circulation^3^. Beginning with the equatorial divergence, the poleward-flowing upper branch of the Subtropical Cells absorb C_ant_ via gas exchange. In transit these waters become denser in stages, culminating with a substantial heat loss in western boundary currents and on the poleward flanks of the Subtropical Cells. This pathway provides a conduit for C_ant_ absorbed in the low latitudes to enter the ocean interior in the subpolar water masses. Both direct overturning and C_ant_ diffusion have proven to be important, consistent with results obtained with kinematic diagnostics of subduction across the mixed layer base^36^.

Although our analysis confirms the importance of the Southern Ocean and the NADW formation region for C_ant_ transport into the ocean interior, our results also highlight the role low latitude accumulation offers in a critical supporting role. Previous models and kinematic diagnostics^12^,^37^ have led to a local characterization of subduction of C_ant_ within SAMW and AAIW. These studies imply injection rates that are consistent with our results, namely that 40% of C_ant_ accumulates in SAMW and AAIW^1^. The insight provided here is the connection between mode water formation and large scale water mass pre-conditioning at lower latitudes. The proposed mechanism, diabatic exchanges between STMW and SAMW, is supported by eddy-permitting models^38^ and data inferences^37^,^39^,^40^. Since water mass transformations are directly impacted by eddy parameterizations and model resolution, the exact numbers may vary but the main processes are represented.

This study has established a framework that links C_ant_ uptake to thermodynamics. The climatological poleward heat transport in the Shallow Overturning Circulation plays a central role. This result motivated our consideration of the more general Shallow Overturning Circulation framework, and the view that more than 50% of SPMW formation sources are subtropical. More generally, the unified STMW/SPMW grouping is best viewed as a binding agent between the subtropical and subpolar dynamical regimes. In turn, these regimes may be expected to experience direct but different impact under climate change. The method developed and applied here is perfectly general, and it is our hope that this method will also be applied to interpret Earth system models to understand how the ocean’s full carbon reservoir will be perturbed under future climate change.

## Methods

### Analysis of GLODAP C_ant_ from WOCE-era measurements

The inventory of anthropogenic carbon^1^ in density space has been derived using the GLODAP data product^1^,^26^. We consider the partitioning of C_ant_ into water masses. We begin with a coarse-grained analysis of the anthropogenic carbon inventory in density space as derived from the ∆C^∗^-based GLODAP data product^1^,^2^, and from the TTD-based product^15^. These estimates have been recently compared^7^. While pattern differences were identified, they remain quite similar in terms of global inventory per water mass in the upper 1000 meters of the water column (see Fig. S2). Since we have chosen to group density layers below intermediate horizons into a single class, we focus only on GLODAP.

We calculated potential density for our reference frame using temperature and salinity climatologies from the World Ocean Atlas 2009 (WOA09)^41^. We have chosen five discrete levels: (i) Tropical Waters (TW) with σ_0_ ≤ 24.5; (ii) lower thermocline waters (STMW) with 24.5 < σ_0_ ≤ 26.6; (iii) sub-arctic and Subantarctic Mode Waters (SAMW) with 26.6 < σ_0_ ≤ 27.1; (iv) Intermediate Waters (AAIW, NPIW) with 27.1 < σ_0_ ≤ 27.4 ; and (v) deep waters (DW) with σ_0_ > 27.4. The separation between TW and STMW at σ_0_ = 24.5 was chosen to match the separation between the equatorially divergent (TW) and convergent (STMW) flows in the Indo-Pacific Subtropical Cells (STCs)^42^.

Inventories and volume-weighted average concentrations were calculated for each density layer. Binning was performed in two ways. First, density binning was performed over the full ocean volume. Second, the same procedure was followed, but the maximum winter mixed layer depth was used to separate the ocean interior from the region directly impacted by seasonal variations. We used a data-derived mixed layer depth climatology^43^ to define the needed levels. We used the 2008 product, that includes Argo data and captures, to first-order, the shape of the maximum wintertime mixed layer in remote regions such as the Southern Ocean.

### The Ocean Carbon Model

The ice-ocean model we used is a nominal two-degree global version of ORCA2-LIM-PISCES^32^ with 31 vertical levels. It includes the classical Gent and McWilliams mesoscale parameterization^44^ with lateral mixing evaluated along isoneutral surfaces. The lateral exchange coefficient depends on the baroclinic instability growth rate^45^. The vertical mixing scheme uses turbulent closure^46^, and there is a diffusive bottom boundary layer parameterization^47^. Background vertical diffusivity increases downward to account for decreased stratification and increased small-scale turbulence near the bottom. Values range from 0.12 10^−4^ m^2^s^−1^ in the top kilometer to 1.2 10^−4^ m^2^s^−1^ at 5000 m. Vertical mixing due to convection was parameterized by locally enhancing vertical diffusivity. Air-sea fluxes of heat and freshwater (evaporation) are evaluated using bulk formulae^48^. A clear-water penetrative short- wave solar radiation formulation was used and surface salinities were restored to climatology. Climatological European Remote Sensing Satellite-1 and -2 scatterometer monthly mean wind stresses were used for the tropics, and the NCEP-NCAR climatological dataset was used poleward of 50°N and 50°S. At the bottom, geothermal heating was represented as a spatially variable heat source.

The model was spun up for 1500 years with climatological repeating seasonal forcing fields. Natural and anthropogenic climate variability were not considered. These fields are identical to those used in previous studies^20^,^21^,^29^. A detailed description of the model and the simulation characteristics are included in those references. From the final spinup year, 15-day means of circulation fields and tendency terms were stored for analysis.

We used the PISCES (Pelagic Inter- action Scheme for Carbon and Ecosystem Studies) model^32^ biogeochemistry with 24 components. Phytoplankton growth can be limited by five different nutrients: nitrate, ammonium, phosphate, silicate and iron. Four living pools are represented: two phytoplankton size classes/groups (nanophytoplankton and diatoms) and two zooplankton size classes (microzooplankton and mesozooplankton). Diatoms differ from nanophytoplankton by their need for Si, by higher requirements for Fe and by higher half-saturation constants because of their larger mean size. For all living compartments, the ratios between C, N and P are kept constant. The internal Fe contents of phytoplankton groups and the Si of diatoms are a function of the external concentrations in nutrients and the light level. The Chl/C ratio is modelled explicitly. All zooplankton elemental ratios are kept constant. There are three non-living components: semilabile dissolved organic matter (with timescales of several weeks to several years), small, and large sinking particles. As with the living components, constant Redfield ratios are imposed for C/N/P while the iron, silicon and calcite pools of the particles are fully simulated and, their ratios are allowed to vary relative to organic carbon. Nutrients are supplied from three sources: atmospheric dust deposition, rivers, and sediment mobilization. The global PISCES configuration has been used to study past climates, to understand the interannual variability in marine productivity or ocean-atmosphere carbon fluxes, to assess the impact of climate change or ocean acidification on marine ecosystems and air-sea carbon fluxes, to evaluate geo-engineering strategies to mitigate climate change.

The biogeochemical model was run using the same constraints as the physical model including lateral mixing on isoneutral surfaces. The model was spun up offline for 3000 years using state variables from the final year of the spinup of LIM-ORCA2. Pre-anthropogenic boundary conditions were maintained for sea surface atmospheric CO_2_ concentrations. This long spinup run was split in 1860. A control run continued through to the end of 1999 with pre-industrial atmospheric CO_2_ concentrations. A second run followed the observed atmospheric CO_2_ transient. As before, we refer to the first run as “natural” carbon and the second run as “contemporary” carbon. The difference in carbon variables between the runs, our focus here, is “anthropogenic carbon”. The modelled C_ant_ inventory compares rather well with GLODAP and TTD estimates. The model has a deep ocean C_ant_ inventory that is higher than GLODAP, but less than TTD. The meridional transport at 30°S also compares well with published oceanic inversions (see Table 1).

## Theory

### Tracer dynamical budget in a water mass framework

Detailed theoretical derivation are presented in the Supplementary Note.

### The conceptual model: assumptions and analytical solutions

We developed a conceptual ocean model having n density layers. This ocean was exposed to an atmospheric tracer boundary condition that grew exponentially with time. The density layers were internally homogeneous.

A steady state ocean circulation implies that the net overturning convergence is zero (volumes neither inflate nor deflate). Here we only considered the subsurface (in the MWML, generally about 100–200 m) expression of the overturning and it is divergent. We discarded the overturning component in the interior since, as illustrated in the GCM analysis, the tracer concentration at depth is much lower than at surface. The drastic assumption of well-mixed layers is at odds with the real ocean, but this simplification contributes to process-attribution. In practice we are assuming that: (1) most of the convergence occurs at the surface, in the mixed layer; (2) the layer specific inventory and air-sea flux properties are controlled not only by the mixed layer but also by the internal volume. This is acceptable especially for layers that are not completely saturated but have a significant vertical exchange (i.e., subduction) and thus for the various mode and intermediate waters which are the focus of this study. We also disregarded the impact of carbonate chemistry, such as solubility effects, and variability in the surface mixed layer thickness.

The resulting equation is solved numerically. For diapycnal transport ω, we used an analytical description of the diapycnal transport that approximately matches the transport in Fig. 4a. For dense water formation (σ_0_ > 27.5) we amplified overturning by a factor 7.5 to compensate for underestimation of surface values for water masses with very large volume. This factor was chosen to represent adiapycnal tracer transport (Supplementary Fig. 3) similar to the C_ant_ overturning in Fig. 4b. Our choice has only a minimal impact on mode and intermediate water masses and on the air-sea fluxes. The two peaks in Fig. 4b correspond to SOC and DW formation (essentially NADW).

Finally, valuable insight was achieved by evaluating analytical solutions, which can be easily derived for the AIRSEA case, that is, here we considered a situation where only air-sea exchange was allowed (β_i_ = 0 and ω_i_ = 0 ∀i). We obtained an analytically result by seeking a solution of the form c_i_ = c_0_ γ_i_ e^t/τatm^.

This yielded


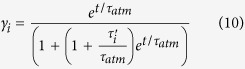


where 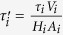 is the relaxation time scale rescaled by the ratio of the water mass volume to the upper boundary layer (*A*_*i*_ *H*_*i*_) volume. For a fast adjustment time scale τ_i_′, γ_i_ = 1 and the water mass concentration perfectly tracks the atmospheric one (c = c_atm_).

The shape factor γ^i^ is also a function of time, but after a relatively short transient period (when e^t/τatm^ ≫ 1) the solution becomes


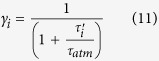


Thus, for all the water masses the carbon content increases exponentially with the same doubling time as the atmosphere. Relative repartition of carbon among the water masses is constant in time and equal to Eq. 11.

Similarly, the surface fluxes increase exponentially:


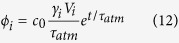


In this instance the repartitioning is not time dependent but rather varies with water masses characteristics (volume, surface area, etc). The surface flux is in fact a function of the product of the water volume, that sharply increases with density, by γ_i_, which is constant for the water masses up to the subtropical densities (τ′ < τ_atm_) and decrease to zero with increasing density. This decrease occurs because the volume strongly increases with density while the surface area is relatively constant for intermediate water masses and goes nearly to zero for the deep ones; Fig. 2). The combination of an increase of the volume and decrease of γ_i_ with increasing density create a maximum of air-sea flux in correspondence of the intermediate water masses that is due only to geometrical factors that in turn depend on oceanic circulation patterns^49^,^50^.

## Additional Information

**How to cite this article**: Iudicone, D. *et al*. The formation of the ocean’s anthropogenic carbon reservoir. *Sci. Rep*. **6**, 35473; doi: 10.1038/srep35473 (2016).

**Publisher’s note**: Springer Nature remains neutral with regard to jurisdictional claims in published maps and institutional affiliations.

## Supplementary Material

Supplementary Information

## Figures and Tables

**Table 1:** Water mass specific GLODAP ΔC* and TTD-based *C*_*ant*_ inventory estimates, volume inventory and outcrop area.

		Total	TW σ_θ_ < 24.5	STMW 24.5 < σ_θ_ < 26.6	SPWM 26.6 < σ_θ_ < 27.0	IW 27.0 < σ_θ_ < 27.5	DW 27.5 < σ_θ_
**(a) WHOLE OCEAN**^**(1)**^							
**Absolute values**							
*C*_*ant*_ inventory	[PgC] - ΔC*^(2)^	104,7	7,5	28,7	24,2	26,3	18,0
*C*_*ant*_ inventory	[PgC] - TTD^(3)^	140,6	8,8	29,5	23,3	27,5	51,4
Volume	[10^18^ m^3^]	1312,3	15,2	64,8	75,1	200,0	957,1
Outcrop area	[10^12^ m^2^]	348,0	164,7	120,9	22,9	35,7	3,7
Winter Outcrop Area (WOA09)	[10^12^ m^2^]	348,5	129,4	129,1	34,9	37,1	18
**Relative fractions**							
*C*_*ant*_ inventory	[%] - ΔC*	—	7,2	27,4	23,1	25,1	17,2
*C*_*ant*_ inventory	[%] - TTD	—	6,3	21,0	16,6	19,6	36,6
Volume	[%]	—	1,2	4,9	5,7	15,2	72,9
Outcrop area	[%]	—	47,3	34,7	6,6	10,3	1,1
Winter Outcrop area	[%]	—	37,1	37,0	10,0	10,6	5,2
**Cumulative fractions**							
*C*_*ant*_ inventory	[%] - ΔC*	—	7,2	34,6	57,7	82,8	100
*C*_*ant*_ inventory	[%] - TTD	—	6,3	27,2	43,8	63,4	100
Volume	[%]	—	1,2	6,1	11,8	27,1	100
Outcrop area	[%]	—	47,3	82,1	88,6	98,9	100
Winter Outcrop area	[%]	—	37,1	74,2	84,2	94,8	100
**(b) MWML ONLY**^**(1,4)**^							
**Absolute values**							
*C*_*ant*_ inventory	[PgC] - ΔC*	18,3	4,8	6,9	2,8	3,0	0,9
*C*_*ant*_ inventory	[PgC] - TTD	19,3	5,7	7,0	2,6	2,9	1,0
Volume	[10^18^ m^3^]	37,3	9,5	12,7	5,4	6,8	2,9
**Relative fractions**							
*C*_*ant*_ inventory	[%] - ΔC*	17.5^(5)^	26,2	37,7	15,3	16,4	4,9
*C*_*ant*_ inventory	[%] - TTD	13.7^(5)^	29,5	36,3	13,5	15,0	5,2
Volume	[%]	2.8^(5)^	25,5	34,0	14,5	18,2	7,8
**Cumulative fractions**							
*C*_*ant*_ inventory	[%] - ΔC*	—	26,2	63,9	79,2	95,6	100
*C*_*ant*_ inventory	[%] - TTD	—	29,5	65,8	79,3	94,3	100
Volume	[%]	—	25,5	59,5	74,0	92,2	100

^(1)^Excluding the Arctic and marginal seas.

^(2)^[1], [2].

^(3)^[4].

^(4)^The bottom of the MWML is defined by the deepest depth of the mixed layer found in the monthly climatology of [9].

^(5)^Fraction relative to whole ocean inventory.

**Table 2:** Summary of the main fluxes controlling the model global anthropogenic CO_2_ inventory (C_ant_).

	Total	TW	STMW	SPWM	IW	DW
**(a) Ocean gain** 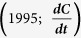		σ_θ_ < 24.5	24.5 < σ_θ_ < 26.6	26.6 < σ_θ_ < 27.05	27.0 < σ_θ_ < 27.55	27.55 < σ_θ_
Inventory [PgC/yr]	2.07	0.16	0.54	0.42	0.52	0.43
Fractions [%]	—	7.7	26.1	20.3	25.1	20.8
Cumulative fractions [%]	—	7.7	33.8	54.1	79.2	100
**(b) Air-sea**						
Fluxes [PgC/yr]	2.04	0.57	0.60	0.36	0.42	0.09
Fractions [%]	—	27.9	29.4	17.6	20.6	4.4
Cumulative fractions [%]	—	27.9	57.4	75.0	95.6	100
**(c) Diapycnal fluxes divergence (**Δ_***overturning***_)						
Global [PgC/yr]	—	−0.36	0.12	0.12	−0.07	0.19
MWML [PgC/yr]	—	−0.47	0.13	0.23	−0.03	0.16
Interior [PgC/yr]	—	0.11	0.00	−0.11	−0.04	0.03
**(d) Air-sea + Diapycnal fluxes (*****ϕ*** + Δ_***overturning***_)						
Global [PgC/yr]	2.04	0.21	0.72	0.48	0.35	0.28
MWML [PgC/yr]	2.04	0.10	0.73	0.59	0.39	0.25
**(e) Residual (a minus d; ~Δ**_***diffusion***_)						
GLOBAL [PgC/yr]	—	−0.05	−0.18	−0.06	0.17	0.15
**(f) Inventory** [PgC]	94.30	8.00	26.00	19.2	23.7	17.4

The values have been estimated using Eq. 4. The diffusive contribution is evaluated as a residual that implies that all kind of diffusive processes are considered, i.e., including any potential numerical diffusion. The coarse-graining of the ocean density structure into five discrete layers is as follows: tropical waters (TW), subtropical mode waters (STMW), subpolar mode waters (SPMW), intermediate water (IW), and deep water (DW). Values are net for each water mass.
