# Supplementary material for: The formation of the ocean’s anthropogenic carbon reservoir
Source: Sci Rep. 2016 Nov 3;6:35473. doi: 10.1038/srep35473 (PMC5093862; doi:10.1038/srep35473)
Supplement: Supplementary Information [file srep35473-s1.pdf]

SUPPLEMENTARY MATERIAL

The formation of the ocean's anthropogenic carbon reservoir

Daniele Iudicone, Keith B. Rodgers, Yves Plancherel, Robert Key, Olivier Aumont, Taka Ito, Gurvan Madec and Masao Ishii

1 Supplementary Note

1.1 Theory

Here we presents in more details the ideas presented above. In practice, the classical water mass diagnostics of the impact of physical terms on the diapycnal fluxes (initiated by[1]; see also[2], and[3]) has been extended to provide a mean for the quantitative analysis of physical *and* biogeochemical processes that rule the tracer inventory in a particular oceanic region.

In other words, the aim is to compute the budget of the divergence/convergence of the tracer due to transport across the isopycnals, i.e., the boundaries of the water masses, separate this into the different processes sustaining the diapycnal transport, and compare with biogeochemical sources/sinks and the tracer diffusive processes. This is given by a very long equation which contains all the the tendency terms for temperature, salinity and the tracer whose derivation is presented in the following. First, we will review the generalized approach for the evaluation of diapycnal transports presented in[4], which introduced the use of neutral density and a term which considers the effect of the penetration of solar radiation into the upper layers of the ocean, a term previously neglected and which is of importance in the tropical and subtropical regions of the oceans.

A schematic for the framework is shown in Fig.3. Given a volume V_γ of water bounded by the ocean bottom, the ocean surface, one isopycnal surface S_γ and an open boundary OB_γ, the scope is first to estimate the volume transport across the isopycnal surface. (Here we assume the volume transport at the surface is negligible even if it is fully considered by the diagnostics,[4].)

The following formulations are valid for any density variable, including neutral density and potential density, where the latter is the one applied here. In the results sections we will use the generic term "density".

The equation of evolution of the density ρ is $D\rho/Dt = d_\rho + f_\rho$ where d_ρ are the mixing processes (vertical and lateral or eddy diffusive processes and bottom layers turbulence) and f_ρ are the boundary buoyancy forcing (air-sea and ice-sea interactions and geothermal flux).

We introduce two scalar quantities, ω_ρ and $|\nabla\rho|$, defined as the velocity across the moving S_ρ and the modulus of the gradient of ρ (by definition it has a direction normal to S_ρ). In this framework, the material derivative of ρ, $\frac{D\rho}{Dt} \equiv \frac{\partial\rho}{\partial t} + \mathbf{u}\cdot\nabla\rho$, becomes simply $\omega_\rho|\nabla\rho|$. We can thus define

$$\omega_\rho = (d_\rho + f_\rho)|\nabla\rho|^{-1} \tag{1}$$

as the diapycnal velocity across the moving S_ρ. Thus, the total volume transport across the isopycnal surface S_ρ becomes

$$\Omega_\rho = \int_{S_\rho} \omega_\rho ds = \int_{S_\rho} (d_\rho + f_\rho)|\nabla\rho|^{-1} ds = \frac{\partial}{\partial\rho}\int_{V_\rho} d_\rho dv + \frac{\partial}{\partial\rho}\int_{V_\rho} f_\rho dv \tag{2}$$

where the latter step was made making use of a generalized form of Leibnitz's theorem[3]. One should note that the lateral (isoneutral) diffusive processes can give rise to net diapycnal transports due to the non-linearity of the equation of state, via processes as cabbeling and thermobaricity. It is also worth noting that only the use of neutral density allows for a correct evaluation of the latter term. In practice, the diapycnal transports are evaluated in terms of non-advective processes and external forcing acting on temperature and salinity.

1.1.1 The tracer evolution

The formulation above will be used here for the evaluation of the tracer transports. Consider the ocean volume delimited by an open boundary, the ocean surface and an isopycnal ρ (Fig. S10) as being filled with a tracer whose inventory (or stock) is $C = \int_{V_\rho} c dv$ where $c = c(x, y, z, t)$ is a local concentration per unit volume. (The results that follows are also valid for a concentration expressed per unit mass.). Assume that the tracer c is transported by the flow, that it experiences diffusive processes d_c and that it is *reactive*, i.e., it has sources and sinks π_c such has that its time evolution is:

$$\frac{Dc}{Dt} \equiv \frac{\partial c}{\partial t} + \mathbf{u} \cdot \nabla c = d_c + \pi_c. \tag{3}$$

First, we express the rate of change of the integrated tracer content as the sum of the integral of the local derivative and the contribution to the inventory from the inflation/deflation of the volume:

$$\frac{dC}{dt} \equiv \frac{d}{dt} \int_{V_\rho} c dv = \int_{V_\rho} \frac{\partial c}{\partial t} dv + \int_{\partial V_\rho} c \mathbf{u}_S \cdot \mathbf{n} ds \tag{4}$$

where $\mathbf{u}_S$ is the velocity of the isopycnal surface and ∂V_ρ is the surface delimiting V (here $\partial V_\rho = S_\rho + \mathrm{OB}_\rho$). Importantly $\frac{d}{dt}$ is used instead of $\frac{\partial}{\partial t}$ as there are no dependence on spatial variables. In other words, it is the time derivative of a volume integral over a time-varying reference volume and thus there are no assumption of steadiness for the water mass volume.

Assuming incompressibility, invoking Gauss's theorem and using Eq. (4), the left hand terms of the integral over the volume V_ρ of Eq. (3) becomes

$$\frac{dC}{dt} + \int_{S_\rho} c \mathbf{u}_r \cdot \mathbf{n} ds + \int_{OB_\rho} c \mathbf{u} \cdot \mathbf{n} ds \tag{5}$$

where $\mathbf{u}_r$ is the velocity relative to the same surface (i.e., $\mathbf{u}_r = \mathbf{u} - \mathbf{u}_S$) and $\mathbf{u}_r = \mathbf{u}$ across OB_ρ.

The quantity that we were looking for is the first term, i.e., the rate of change of the extensive quantity C within the volume of water denser than ρ. The second term represents the transport across the isopycnal surface in the framework moving with the isopycnal and the last one consists in the transport at the open boundary.

Importantly, the dianeutral transport of the tracer Φ_ρ can be expressed in terms of the ocean physics, i.e., the processes that act on transforming water masses, here divided into mixing processes (d_ρ) and boundary forcing (f_ρ). Using $\omega_\rho = (d_\rho + f_\rho)|\nabla\rho|^{-1}$ (Eq. 1) as dianeutral velocity relative the moving ∂V_ρ, the transport of c per unit surface across a neutral density surface is

$$\phi_\rho = c\omega_\rho = c(d_\rho + f_\rho)|\nabla\rho|^{-1}. \tag{6}$$

In fact, the tracer flux Φ_ρ across the S_ρ is

$$\begin{aligned}\Phi_\rho \equiv \int_{\partial V_\rho} c\omega_\rho ds = \int_{\partial V_\rho} c(d_\rho + f_\rho)|\nabla\rho|^{-1} ds \\ = \underbrace{\frac{\partial}{\partial\rho}\int_{V_\rho} c d_\rho dv}_{\text{Diffusive buoyancy processes}} + \underbrace{\frac{\partial}{\partial\rho}\int_{V_\rho} c f_\rho dv}_{\text{Surface buoyancy processes}}\end{aligned} \tag{7}$$

where the latter passage was made making use of a generalized form of Leibnitz's theorem. Importantly, the product in (6) is done locally on the isopycnal surface, i.e., before evaluating the integrals.

Considering the equivalence $\omega_\rho \equiv \mathbf{u}_r \cdot \mathbf{n}$ and the expression above, the expression for the rate of change of the extensive tracer value becomes

$$\begin{aligned}\underbrace{\frac{dC}{dt}}_{\text{Tracer rate of change}} = -\underbrace{\left[\frac{\partial}{\partial\rho}\int_{V_\rho} c d_\rho dv + \frac{\partial}{\partial\rho}\int_{V_\rho} c f_\rho dv\right]}_{\text{watermass transformation}} \\ - \underbrace{\int_{BV_\rho} c\mathbf{u}\cdot\mathbf{n} ds}_{\text{Transport at boundary}} + \underbrace{\int_{OS_\rho} \phi_a ds}_{\text{Air-sea exchange}} + \underbrace{\int_{V_\rho} d_c dv}_{\text{Diffusion}} + \underbrace{\int_{V_\rho} \pi_c dv}_{\text{Internal sources/sinks}} .\end{aligned} \tag{8}$$

By considering the tendency terms for temperature and salinity[4] and for the tracer field c all the terms in Eq. (8) can be easily evaluated. In fact, the diapycnal transport is computed

by evaluating the derivative of the volume integral of the tendency terms of density using a binning procedure[4].

In the following we presents to extreme cases for easing the understanding of the general value of the above expression:

1. In the case of steady state buoyancy processes and transient tracer dynamics we obtain $\mathbf{u}_S = 0$. Therefore the rate of change of the tracer inventory (left hand term in Eq. 8) is simply

$$\frac{dC}{dt} \equiv \int_{V_\rho} \frac{\partial c}{\partial t} dv \tag{9}$$

For the very idealised case of a purely advected tracer (a passive tracer with zero sources and sinks and zero diffusion) from Eq. (8) we have that the rate of change of the tracer inventory is equal to the convergence of the tracer produced by the diapycnal transport at the (fixed) boundaries (i.e., by advection), as expected intuitively.

2. In the case in which the tracer is constant in space but the water mass volume changes we have

$$\frac{dC}{dt} \equiv \int_{\partial V_\rho} c\mathbf{u}_S \cdot \mathbf{n} ds \tag{10}$$

which can be expressed as the sum of the thermodynamical processes acting on the water mass and the convergence of the lateral transport at the basin boundary. In the case of still water (null velocity field), with no processes acting on the tracer evolution, while thermodynamic processes are present the isopycnal surface changes in response to the change in density and thus the tracer inventory naturally changes due to the volume variation. This is typically the case of a sunny weather over a calm sea, where the surface layers warm up while retaining their tracer content, de facto causing a net diapycnal transport of the tracer. In this case Eq. (8) simply becomes

$$\frac{dC}{dt} = \underbrace{-\frac{\partial}{\partial \rho} \int_{V_\rho} c(d_\rho + f_\rho) dv}_{\text{Buoyancy processes}} \tag{11}$$

i.e., the effect of the inflating/deflating of the isopycnal volume by thermodynamic processes (e.g., heating or cabbeling) is fully retained (remind that beacuse of $\mathbf{u} = 0$, $\mathbf{u}_r = -\mathbf{u}_S$).

References

1. Walin G. (1982) On the relation between sea-surface heat flow and thermal circulation in the ocean. *Tellus* 34:187-195, doi:10.1111/j.2153-3490.1982.tb01806.x

2. Nurser A.J.G., Marsh R., Williams R.G. (1999) Diagnosing Water Mass Formation from AirSea Fluxes and Surface Mixing. *J. Phys. Oceanogr.* 29:1468-1487.

3. Marshall J., Jamous D., Nilsson J. (1999) Reconciling thermodynamic and dynamic methods of computation of water-mass transformation rates. *Deep Sea Research Part I: Oceanographic Research Papers*, 46(4):545–572.

4. Iudicone D., Madec G., McDougall T.J. (2008) Water-Mass Transformations in a Neutral Density Framework and the Key Role of Light Penetration. *J. Phys. Oceanogr.* 38:1357-1376.

5. Key R.M., al. (2004) A global ocean carbon climatology: Results from Global Data Analysis Project (GLODAP). *Global Biogeochem. Cycles* 18:GB4031, DOI:10.1029/2004GB002247.

6. de Boyer Montégut C., Madec G., Fischer A., Lazar A., Iudicone D. (2004) Mixed layer depth over the global ocean: An examination of profile data and a profile-based climatology. *J. of Geophys. Res. Oceans* 109:C12

7. DeVries, T. (2014) The oceanic anthropogenic CO2 sink: Storage, airsea fluxes, and transports over the industrial era. *Global Biogeochem. Cycles* 28(7):631-647.

2 Supplementary Figures

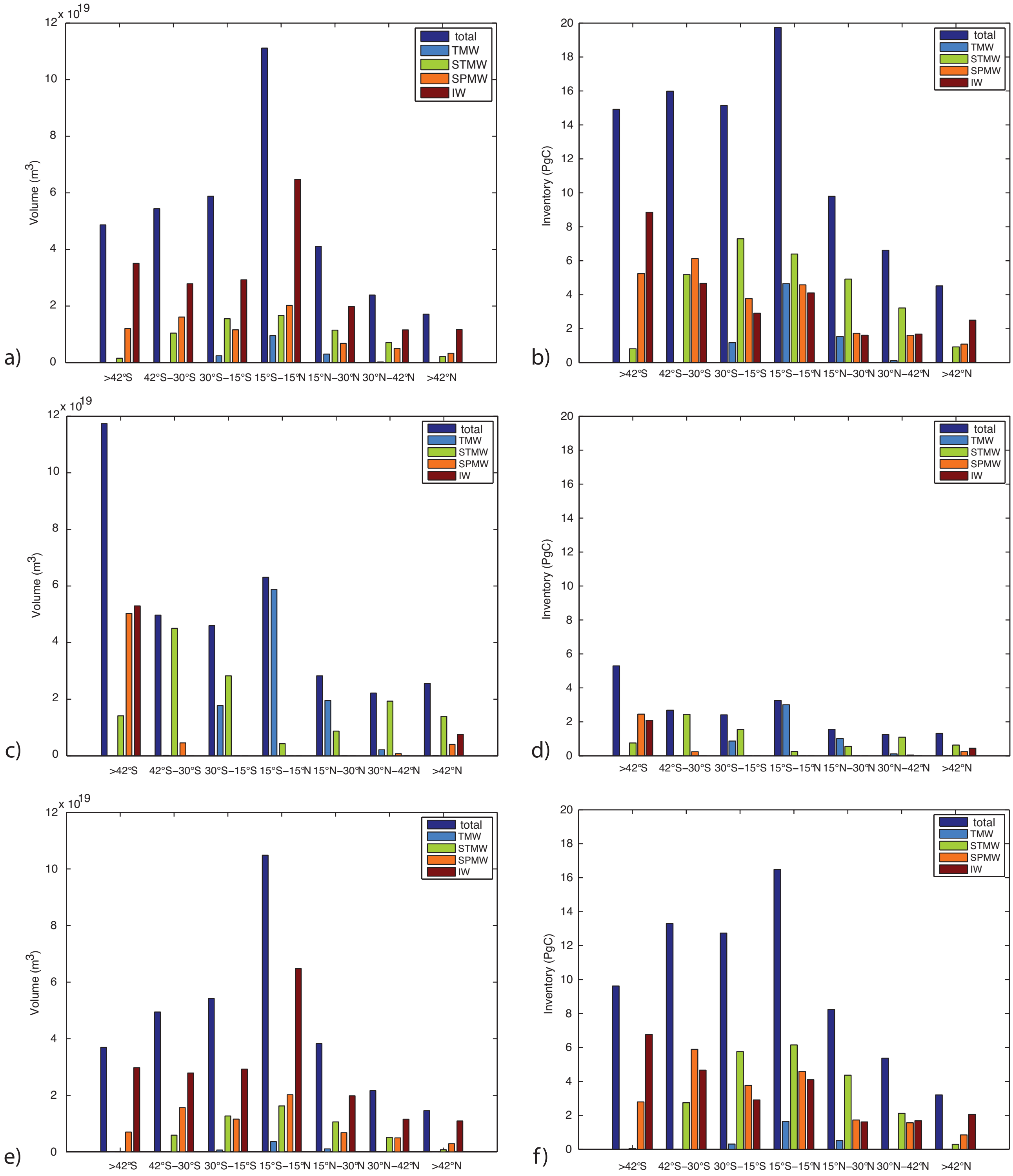

Figure S1: **WOCE-era distribution of C_{ant} in the upper layers of the ocean from the GLODAP product**[5], where WOA09-calculated densities have been used to coarse-grain the density layers. a) Global water masses volumes; b) Global water masses inventories; c) MWML volumes; d) MWML inventories; e) Volumes for the interior; f) Inventories for the interior. Here for MWML it is intended the upper layer above the mixed layer maximum depth (derived from[6]).

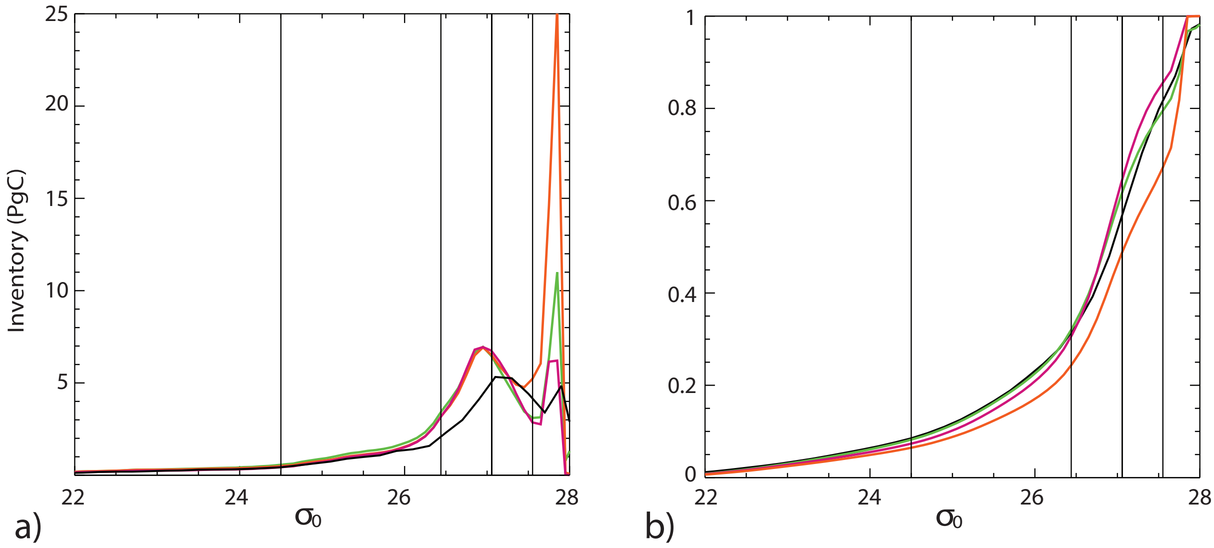

Figure S2: C_{ant} **inventories (binned in 0.1** kg/m^3**).** a) values per water mass; b) cumulative values per water mass. Colors code: GLODAP in red, TTD in orange, data from[7] in green and the numerical model in black.

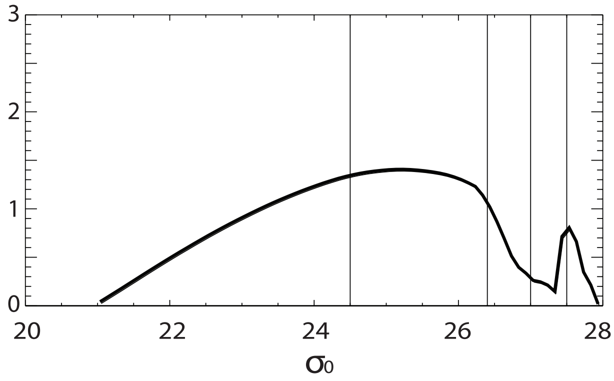

Figure S3: C_{ant} **transformation fluxes Φ_ρ as predicted by the conceptual model** (similarily to Fig. 4b). Values in the plot (units are $c\,m^3/s$, intended as transport of a generic tracer c) have to be multiplied by 10^9 .
